# Effects of photobiomodulation and caffeine treatment on acute kidney injury in a hypoxic ischemic neonatal rat model

**DOI:** 10.14814/phy2.15773

**Published:** 2023-08-07

**Authors:** A. M. Groves, C. J. Johnston, G. Beutner, J. E. Dahlstrom, M. Koina, M. O'Reilly, B. Marples, G. Porter, P. D. Brophy, A. L. Kent

**Affiliations:** ^1^ Department of Radiation Oncology University of Rochester School of Medicine and Dentistry Rochester New York USA; ^2^ Department of Pediatrics University of Rochester School of Medicine and Dentistry Rochester New York USA; ^3^ Department of Pediatrics, Division of Cardiology University of Rochester School of Medicine and Dentistry Rochester New York USA; ^4^ Department of Anatomical Pathology Canberra Hospital Woden Australian Capital Territory Australia; ^5^ College of Health and Medicine, Australian National University Canberra Australian Capital Territory Australia; ^6^ Department of Neonatology, Women's and Babies Division Women's and Children's Hospital Adelaide South Australia Australia; ^7^ University of Adelaide, School of Medicine Adelaide South Australia Australia

## Abstract

Hypoxic ischemic encephalopathy (HIE) occurs in 2–5/1000 births, with acute kidney injury (AKI) occurring in 40%. AKI increases morbidity and mortality. Caffeine, an adenosine receptor antagonist, and photobiomodulation (PBM), working on cytochrome c oxidase, are potential treatments for AKI. To examine effects of caffeine and PBM on AKI in rats, Day 7 pups underwent a HIE intervention (Modified Rice–Vannucci model) replicating pathology observed in humans. Caffeine was administered for 3 days and/or PBM for 5 days following HIE. Weights and urine for biomarkers (NGAL, albumin, KIM‐1, osteopontin) were collected prior to HIE, daily post intervention and at sacrifice. Both treatments reduced kidney injury seen on electron microscopy, but not when combined. HIE elevated urinary NGAL and albumin on Days 1–3 post‐HIE, before returning to control levels. This elevation was significantly reduced by PBM or caffeine. KIM‐1 was significantly elevated for 7 days post‐HIE and was reduced by both treatments. Osteopontin was not altered by HIE or the treatments. Treatments, individually but not in combination, improved HIE‐induced reductions in the enzymatic activity of mitochondrial complexes II‐III. PBM and caffeine also improved weight gain. PBM and caffeine reduces AKI diagnosed by urinary biomarkers and confirmed by EM findings.

## 1 INTRODUCTION

Perinatal asphyxia is a major cause of morbidity and mortality, occurring in 2–5 per 1000 live births (Pfister & Soll, 2010). Multiple organ injury can occur with cardiac dysfunction, acute kidney injury (AKI), hepatic dysfunction (disseminated intravascular coagulopathy) and neurological injury including seizures and hypoxic ischemic encephalopathy (HIE). AKI occurs in up to 40% of neonates with HIE and is an independent risk factor for increased duration of ventilation, length of stay, poor neurodevelopmental outcome, and mortality (Karlowicz & Adelman, 1995; Kirkley et al., 2019; Sarkar et al., 2014; Selewski et al., 2013).

Theophylline (a methylxanthine) has been shown to reduce AKI occurring in term neonates who have suffered a hypoxic ischemic event (Bhatt et al., 2019; Chock et al., 2021). Caffeine (another methylxanthine) has not been studied in term infants following HIE, but has been associated with a reduction in the incidence of AKI in very low birth weight infants (Carmody et al., 2016; Harer et al., 2018). Caffeine is a nonselective adenosine receptor antagonist. Following ischemic renal tubular injury the glomerular adenosine A_1_ receptor is activated causing afferent arteriole vasoconstriction resulting in reduced glomerular filtration rate (GFR) and water retention (Bauerle et al., 2011). Caffeine may ameliorate this effect.

670 nm red light is absorbed by cytochrome c oxidase, the rate limiting enzyme in the terminal phosphorylation of the mitochondria (Tafur & Mills, 2008). It has been shown to reduce the extent of oxygen induced retinal neovascularization, decrease pulmonary hemorrhage and improve survival in rodent models (Natoli et al., 2013). 670 nm photobiomodulation promotes wound healing in skin and oral mucosa (Eells et al., 2004) and reduces cerebral pathology in animal models of brain damage and in human ischemic stroke (Lampl et al., 2007; Oron et al., 2006).

Mitochondrial dysfunction has been shown to occur in AKI and mitochondria‐targeted antioxidants have the potential to protect renal tissue from ischemic effects reducing potential AKI (Jankauskas et al., 2016; Szeto et al., 2017). By reducing mitochondrial energy failure there is the potential to reduce programmed cell death and superoxide radicals. 670 nm PBM is safe and feasible to use in the neonatal population (Kent et al., 2020).

The aim of this study was to examine the effects of PBM, using 670 nm red light, and caffeine on kidney dysfunction following a hypoxic ischemic event in a neonatal animal model. We hypothesized that caffeine and 670 nm red light would reduce evidence of AKI.

## 2 METHODS

### 2.1 Animals

This study was approved by the University of Rochester, University Committee on Animal Resources (102314/2019‐30). Care and handling of the animals was conducted in accordance with the National Institutes of Health guidelines. Pups were obtained from timed‐pregnant Sprague Dawley rats purchased from Charles River Laboratories and were conducted with protocols approved by the Institutional Animal Care and Use Committee (IACUC) of the University. Experimental conditions were randomized within each litter. As such, each experimental group (i.e., HIE or control intervention and treatments) consisted of four to six pups and was comprised of pups from multiple litters and equal numbers of males and females.

### 2.2 Hypoxia‐ischemia intervention

Surgery and hypoxia were performed on 7‐day‐old rat pups to induce HIE (Groves et al., 2022). Two percent isoflurane was used for anesthesia, and subdermal buprenorphine was provided for analgesia. The rat pups underwent the Modified Rice–Vannucci model which consisted of ligation of the left common carotid artery, 1 h recovery then placement in a hypoxia chamber at 8% oxygen for 120 min. Isothermal pads were used to maintain temperature throughout at 37°C. Pups were then returned to home cages to recover and feed as normal. Controls included a no surgery or anesthesia group as well as a sham group that underwent sham surgery with anesthesia to expose the carotid. Weights were recorded daily for the first 3 experimental days and on day of sacrifice.

### 2.3 Treatment regimens

**FIGURE 1:**
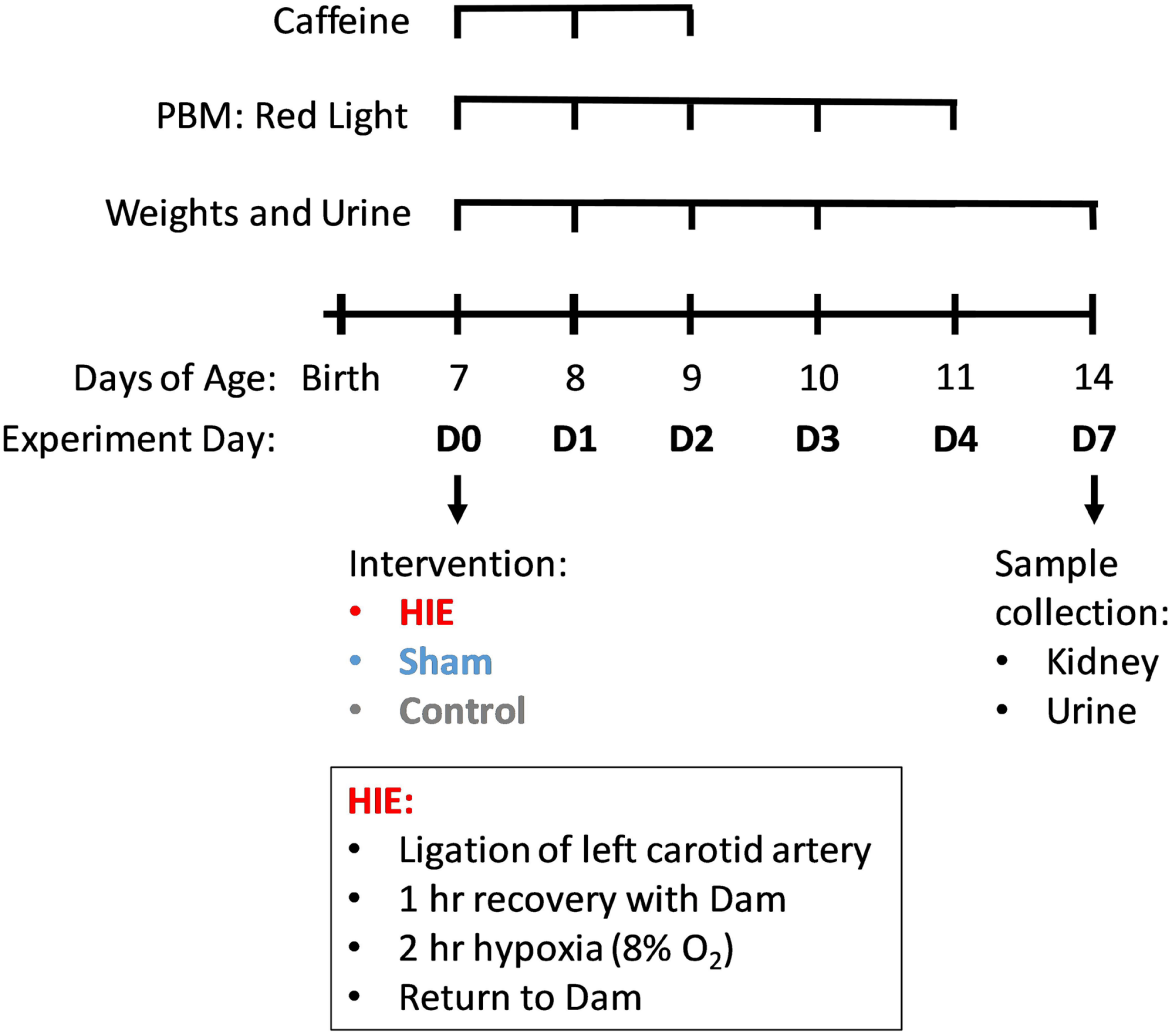
Rice–Vannucci model of HIE and sample collection schedule. HIE: Sprague Dawley rat pups (7 days of age) underwent ligation of the left carotid artery, recovered with their dam for 1 h, then were exposed to hypoxia (8% oxygen for 120 min). Control: no surgery or anesthesia. Treatments: Pups were treated with PBM by 670 nm red light for 5 days or 10 mg/kg caffeine for 3 days, or received both treatments, starting immediately following the HIE procedure. Sample collection: Weights were collected daily. Urine was collected prior to HIE intervention on D0, post intervention on D1‐3, and at sacrifice, D7. Kidneys were collected on D7.

For each treatment group, pups were randomly assigned one of the following treatments: PBM with 670 nm red light, caffeine, both PBM and caffeine, or no treatment (Figure 1). Treatments were initiated directly following the conclusion of the HIE procedure. PBM was conducted immediately prior to caffeine administration in the combined treatment group. Pups receiving PBM were exposed to 670 nm red light at a distance of 3 cm (providing 30 J/cm^2^) using a custom‐built clinical light therapy device (Multi Radiance Medical). Pups were initially exposed for 10 min, then subsequent exposures were administered twice daily for 5 min, spaced 4 h apart, for 4 additional days. Caffeine treatment was administered orally and consisted of 10 mg/kg/dose caffeine citrate (AuroMedics Pharma LLC) diluted in sterile water. Caffeine treatment was continued for an additional 2 days. Pups in control and sham groups also underwent identical treatments as those in the HIE group.

### 2.4 Urine collection and biomarker analysis

Urine was collected, as previously described (Groves et al., 2022), on the day before intervention, on experimental Days 1–3 and on the day of sacrifice, D7. Urine was centrifuged at 300 × ***g*** to remove particulates, filtered with a 0.2‐micron sterile filter and frozen at −80°C. Meso Scale Discovery Rat Kidney Injury Panel V Plex Assay (catalog no. K15162C; Meso Scale Discovery) was used to perform a multiplex ELISA on the urine. Assays were performed according to manufactures instructions. Neutrophil gelatinase‐associated protein (NGAL), albumin, kidney injury molecule (KIM)‐1 and osteopontin were detected and quantified.

### 2.5 Sample processing and electron microscopy

Pups were sacrificed with Euthasol (100 mg/kg; Virbac) followed by exposure of abdominal and chest organs by midline incision at experimental Day 7, corresponding to postnatal Day 14. In one cohort, left kidneys were removed and snap‐frozen in liquid nitrogen. In another cohort, heparin sodium (1 unit/gram body weight), papaverine hydrochloride (1.2 mg dose) and 0.9% sodium chloride was injected into the right atrium to clear vasculature. 2.5% glutaraldehyde in 0.1 M phosphate buffer was then used to perfusion fix tissues. Left kidney cortex pieces approximately 2–3 mm in length and diameter were cut and immersed in 2.5% glutaraldehyde, then postfixed in 1.0% osmium tetroxide and infiltrated in Spurr epoxy resin.

### 2.6 Mitochondrial assessment

Mitochondrial fractions were enriched from frozen kidneys by differential centrifugation. Kidneys were minced and then homogenized in an isotonic buffer (210 mM mannitol, 65 mM sucrose, 20 mM Tris [pH 7.4], 1 mM EGTA and 1 mM EDTA). Homogenates were centrifuged (500 × ***g***, 10 min) then supernatants transferred into fresh microtubes were centrifuged twice with washing (12,000 × ***g***, 10 min) to sediment the mitochondria, and resuspended in isotonic buffer then aliquoted for colorimetric assays and western blots. Complex III was measured by its ability to accept electrons from complex I or II (NADH‐cytochrome c reductase, succinate‐cytochrome *c* dehydrogenase; 550 nm, ε for cytochrome *c* 18.7 mM^−1^ cm^−1^). Protein in samples was determined using a BCA assay kit (Pierce).

### 2.7 Statistics

Statistical analyses were performed on GraphPad Prism Version 9.4.0 (GrpahPad Software LLC). To determine how HIE induced AKI biomarker abundance in urine, for each individual animal the concentration of each biomarker protein was compared before and after the HIE or control intervention by calculating the fold change in protein concentration at each time point from Day 0. For all assays, data are presented as mean ± SEM and statistical significance was determined by two‐way ANOVA. Statistical significance was analyzed for intervention, timepoint and/or treatment comparisons, as indicated in figure legends. When ANOVA showed significant differences a Sidak multiple comparison test was performed. A value of *p* < 0.05 was considered significant.

## 3 RESULTS

### 3.1 PBM and caffeine abrogate pathological evidence of HIE‐Induced AKI

**FIGURE 2:**
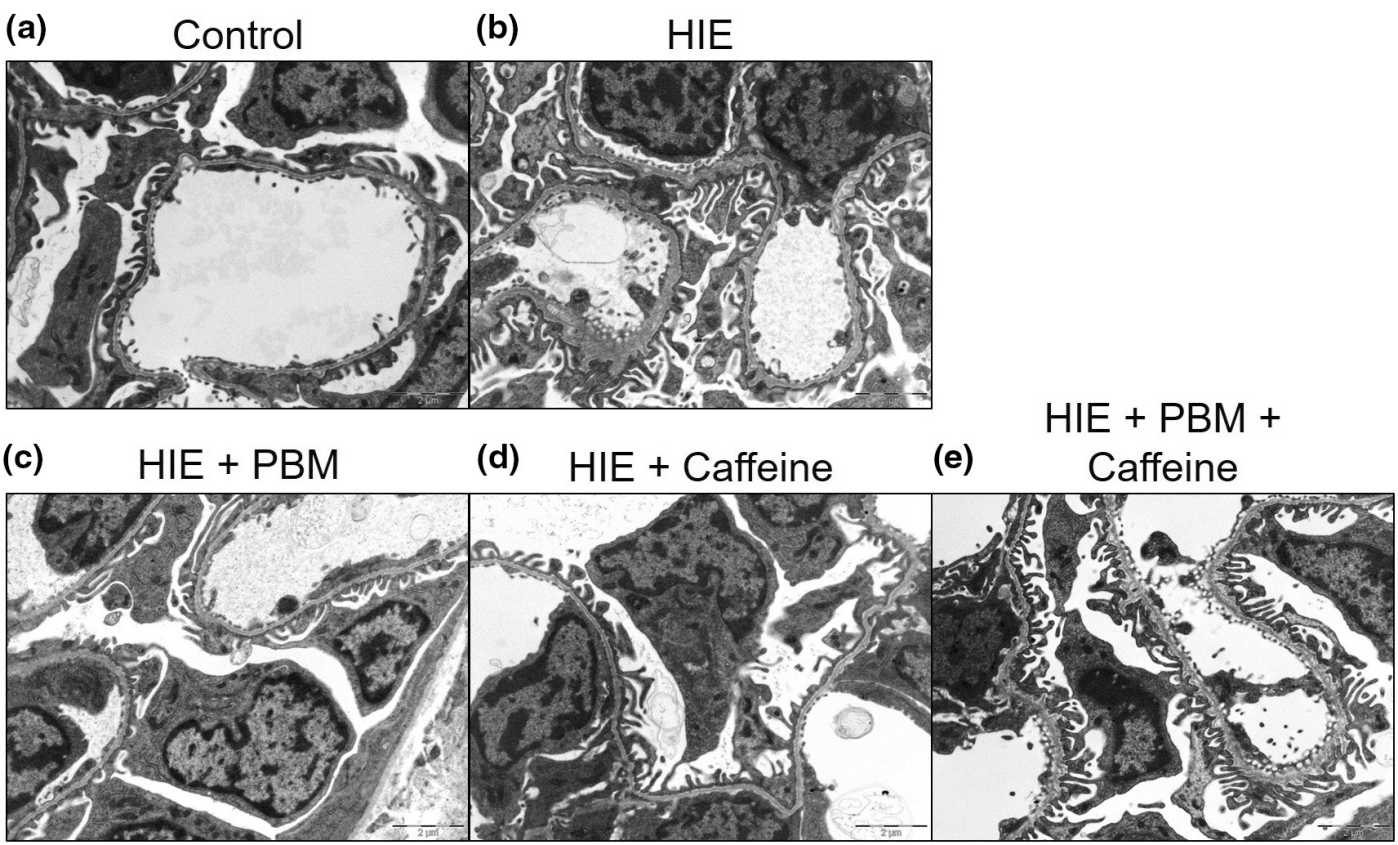
Electron microscopy images of kidneys from rat pups on Day 7 following HIE intervention. Compared to control animals (a), significant changes to the glomerular basement membrane were observed in pups receiving HIE with no treatment (b), while only minor changes were noted in pups receiving either PBM (c) or caffeine alone (d), but not in combination (e). Panels depict representative images of *n* = 3–6 pups/group. Scale bar = 2μm.

A modified Rice–Vannucci model was used to induce HIE in 7 day rat pups, which were subsequently treated with either caffeine, PBM, or both (Figure 1). In order to assess extent of AKI following HIE, and determine whether PBM and caffeine modulated this outcome, kidneys were observed by electron microscopy (EM) and the nature and extent of pathological findings were evaluated (by JED an anatomical pathologist) (Figure 2). EM assessment revealed normal glomerular basement membrane (GBM) and tubular appearances from pups in the control groups (Figure 2a). Significant pathological changes were present in kidneys from pups receiving HIE without any follow‐up treatment (Figure 2b). This included fibrosis a focal inflammatory cell response, increased protein droplets in lysosomes of the proximal tubules and myelinated figures. The GBM also showed significant changes with spikes, laminations and splits present. By contrast, in kidneys from pups that received PBM following HIE intervention there were very few changes to the GBM and the tubules appeared normal (Figure 2c). Similarly, pups that received caffeine treatment post HIE had minimal changes (Figure 2d). These included minor bumps and laminations in the GBM, some vacuolization in the proximal tubules and an increase in fibroblasts in the interstitium. Combination treatment with both PBM and caffeine, however, did not improve HIE‐induced kidney pathology and similar significant pathological changes were observed to those without treatment following HIE intervention (Figure 2e). This included significant spikes, laminations and splits in the GBM, blebs in the distal tubules and loss of microvilli in proximal tubules. Semiquantitative scoring of kidney pathology is provided in Table S1.

### 3.2 PBM and caffeine alleviate HIE‐induced elevations in urinary biomarkers of AKI

**FIGURE 3:**
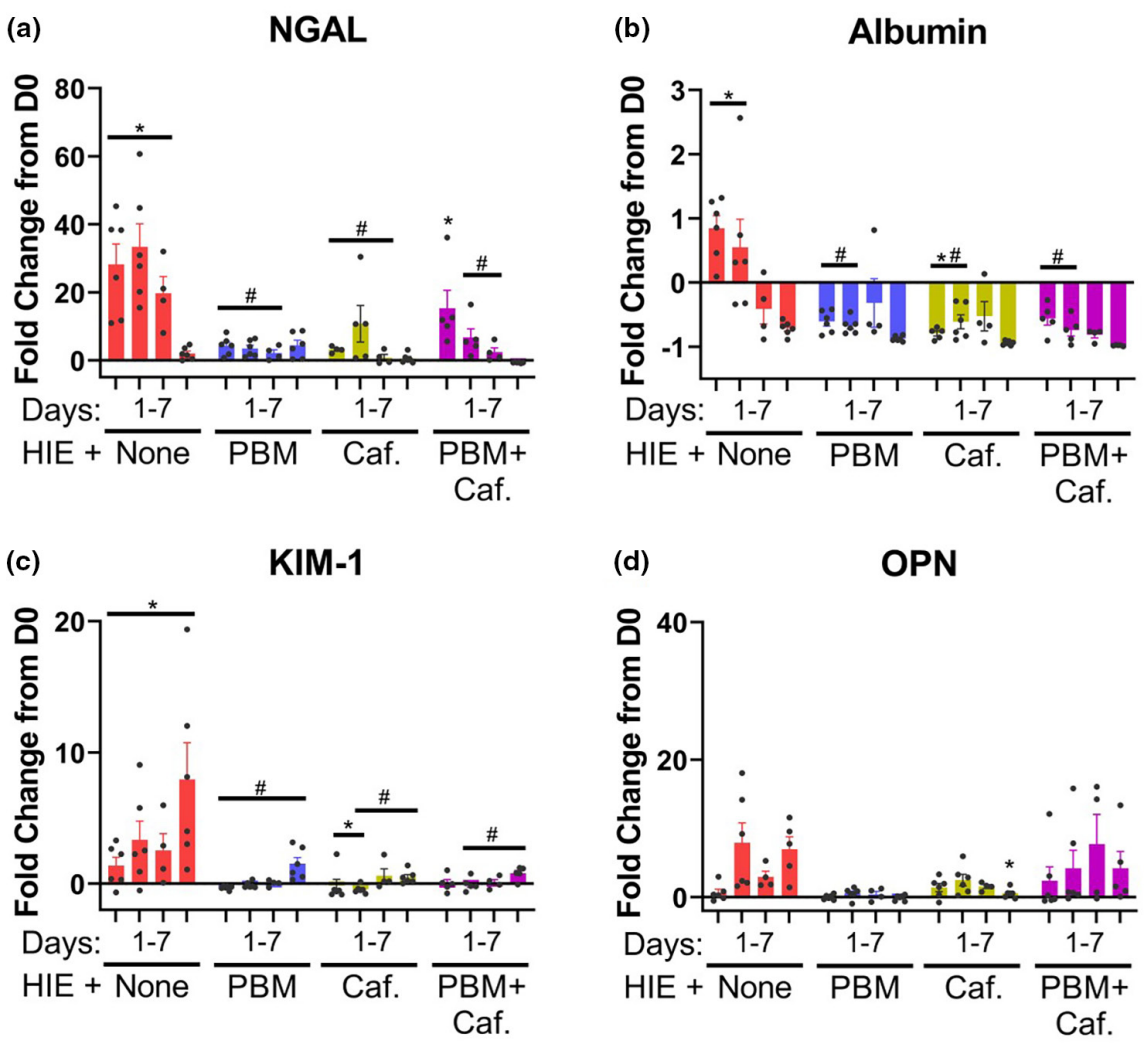
The fold change from D0 within each subject in urinary biomarkers of AKI, collected from pups receiving HIE injury and either no treatment or treatment with PBM or caffeine, alone and in combination, at 1, 2, 3, and 7 days from start of experiment. Protein abundance in urine was determined via ELISA. Bars represent mean ± SD of *n* = 4–6 pups/group. Data points represent individual animals. *Significantly different (*p* < 0.05) from time point matched controls not receiving HIE (control data is presented in Figure S1). ^#^Significantly different (*p* < 0.05) from time point matched HIE pups not receiving PBM or caffeine treatment.

Because recent publications by our group and others have established that the Rice–Vannucci model produces AKI (Groves et al., 2022; Wang et al., 2019; Xu et al., 2017), a panel of recognized urinary biomarkers of AKI were analyzed (fold change from day 0) to assess temporally the effect of PBM and caffeine treatments on the development of AKI (Figure 3). HIE injury elevated NGAL on Days 1–3 (Figure 3a), and Albumin on days 1–2 post HIE intervention (Figure 3b), both of which returned to baseline levels by Day 7. In contrast KIM‐1 was elevated for the duration of the experiment, from Days 1 to 7 post HIE injury (Figure 3c). Both PBM and caffeine treatments significantly reduced elevations in these urinary biomarkers. From 1 to 3 days post HIE intervention, NGAL was significantly lower in pups treated with either PBM or caffeine than in pups that did not receive any treatment, while combination treatment only reduced these values on Days 2–3 (Figure 3a). All treatments significantly reduced Albumin on Days 1–2 (Figure 3b), while KIM‐1 was reduced by PBM on Days 1–7 and by caffeine and combination treatment on Days 2–7 (Figure 3c). Osteopontin was not responsive to either HIE intervention or to the treatments, either alone or in combination on any of the days assayed (Figure 3d). Treatments did not significantly alter urinary biomarker levels in control pups not receiving HIE injury (Figure S1).

### 3.3 HIE reduces kidney mitochondrial electron transport chain complex III activity

**FIGURE 4:**
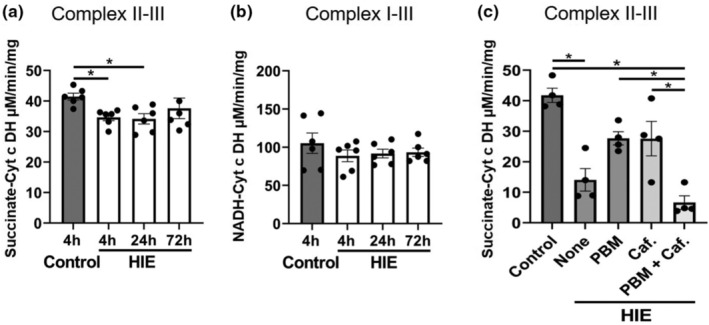
Succinate‐cytochrome *c* dehydrogenase (complex II‐III) and NADH‐cytochrome *c* dehydrogenase (complex I‐III) activity in mitochondria isolated from pups receiving HIE injury and either no treatment or treatment with PBM or caffeine, alone and in combination, at 4, 24, and 72 h (a, b) and 4 h (c) from the start of the start of experiment. Bars represent mean ± SD of *n* = 4–6 pups/group. Data points represent individual animals. *Significantly different (*p* < 0.05) comparisons indicated by brackets.

Following an ischemic event, low oxygen availability can affect mitochondrial ATP generation and damage energy metabolism pathways due to oxidative stress, further contributing to AKI. We investigated the contribution of this mechanism to kidney outcomes by assessing the effects of HIE injury on electron transport complex function in kidney mitochondria (Figure 4). Transient decreases in the ability of complex III to accept electrons from complex II were observed 4–24 h post HIE, which were resolved by 72 h (Figure 4a). This effect was specific to the succinate‐cytochrome *c* dehydrogenase activity, since electron transfer from complex I to III (NADH‐cytochrome *c* dehydrogenase) was not significantly affected (Figure 4b). Further examination of the complex II‐III activity revealed that treatment with either PBM or caffeine, individually rescued the effects of HIE on complex II‐III activity, however the combination of PBM and caffeine did not produce beneficial changes, as observed by EM.

### 3.4 Systemic findings

**FIGURE 5:**
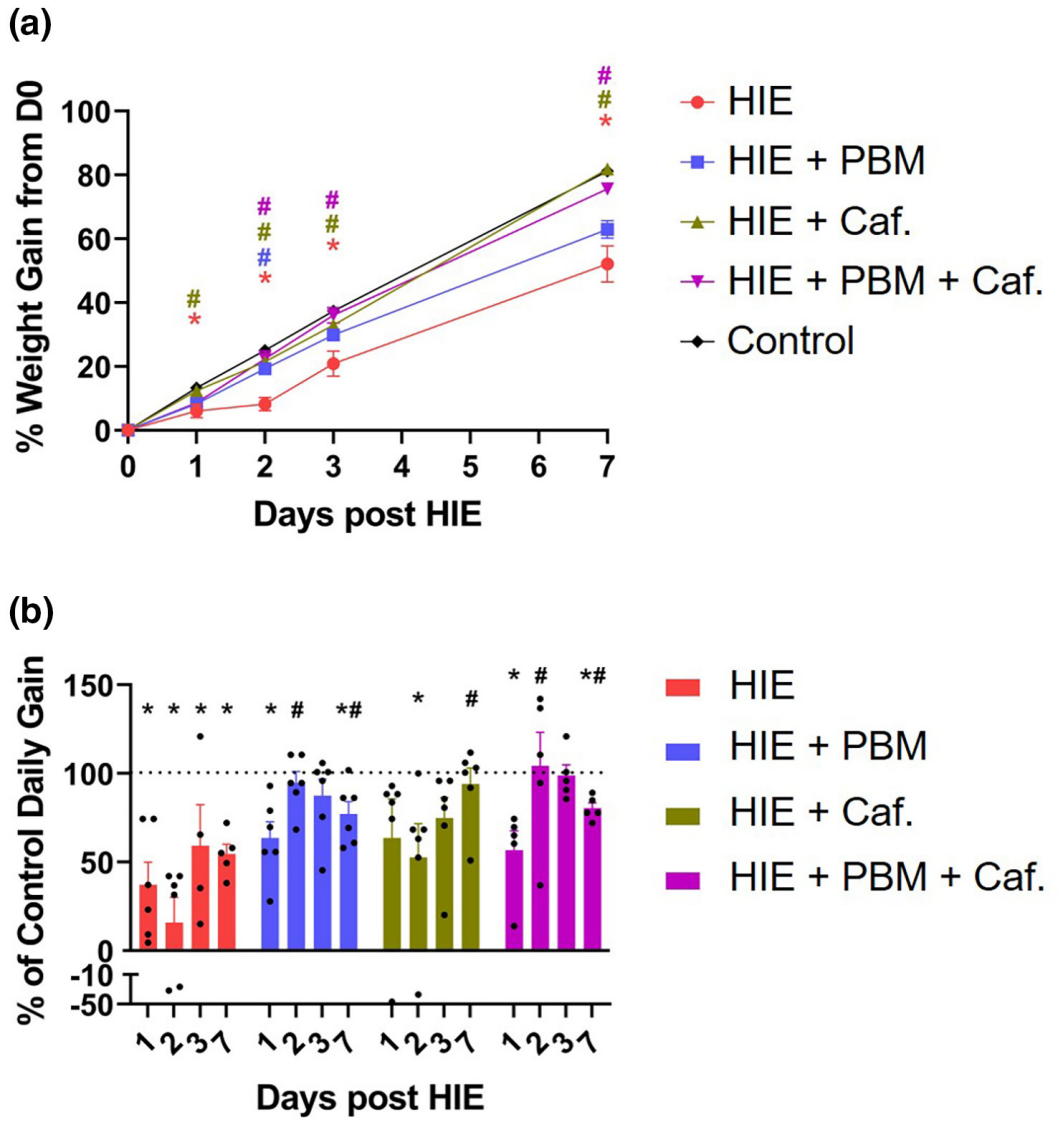
Percent body weight gain above D0 body weight (a) and daily weight gain as percentage of weight gain of control pups (b) of pups receiving HIE injury and either no treatment or treatment with PBM or caffeine, alone and in combination, at 1, 2, 3, and 7 days from start of experiment. Negative values indicate weight loss. Symbols and bars represent mean ± SD of *n* = 4–6 pups/group. Data points represent individual animals. *Significantly different (*p* < 0.05) from time point and treatment matched controls not receiving HIE. ^#^Significantly different (*p* < 0.05) from time point matched HIE pups not receiving treatment.

In order to assess the effect of the treatments on overall health of the animals, body weight was recorded prior to HIE injury and on experimental Days 1–3, and 7 post HIE induction (Figure 5). HIE intervention significantly decreased weight gain in pups to 52.1% above starting weight when compared to controls, which were 81.2% above starting weight, as previously reported (Groves et al., 2022). PBM, caffeine treatment and combination treatment all improved weight gain to 63.0%, 81.2%, and 75.6% above starting weights, respectively (Figure 5a). Daily weight gain was reduced throughout the time course of the study in HIE injured pups not receiving treatment (Figure 5b), but treatment with PBM, with or without caffeine, improved daily weight gains as early as 48 h post HIE induction (Figure 5b). No significant reduction in weight gain was observed in pups receiving sham surgery or in treatment controls without HIE (data not shown). For each assay (Figures 2, 3, 4, 5), data from male and female pups were compared and no significant sex differences were observed (data not shown).

## 4 DISCUSSION

This study has shown that 670 nm red light and caffeine reduce AKI sustained in an HIE injury model. The Rice–Vannucci model has been a useful tool to characterize and interrogate the mechanisms that result in encephalopathy following hypoxic–ischemic injury. Less well studied and only relatively recently reported is the AKI that is also generated in this model. Xu et al. (2017) investigated the role of edema in the pathophysiology of hypoxic ischemic injury, and described histological evidence of kidney injury within 72 h of the procedure. Wang et al. (2019) showed kidney changes in several proteins related to carnitine homeostasis and energy metabolism 24 h after the procedure. We recently reported that injury to the proximal tubules of the kidney was histologically detectable 7 days following insult and was preceded by elevations in urinary biomarkers of AKI (Groves et al., 2022). Our current findings extend this characterization to show that GBM injury, as well as inflammation and fibrosis, is evident on EM imaging 7 days following HIE intervention. Additionally, as demonstrated previously, kidney pathology was preceded by elevations in urinary biomarkers of AKI. Each varied in the extent and timing of elevation, possibly reflecting their differing sensitivity to the various pathologic processes that occur during the onset of kidney injury. Furthermore, we found that this injury was associated with changes in mitochondria function.

Although therapeutic hypothermia is a standard and commonly used therapeutic strategy to manage HIE for neonates, recent studies report that the benefit of this treatment is limited (Mathew et al., 2022). Moreover, studies also indicated that AKI remained a complication in those that received this treatment (Robertsson Grossmann et al., 2022). Studies considering alternative approaches, such as adjuvant therapies have also yielded mixed conclusions (Lee et al., 2019; Nair & Kumar, 2018). Additionally, these reviews considered the neuroprotective effects of these treatments and did not include kidney outcomes. One promising therapy is the use of adenosine receptor antagonists such as caffeine, theophylline and aminophylline, which have found to be effective agents to treat AKI, as they counteract hemodynamic changes in the kidney (Rabadi & Lee, 2015). Activation of adenosine receptors in the glomerulus following ischemic renal tubular injury results in afferent arteriole vasoconstriction, reduced GFR and water retention, which is prevented by these receptor antagonists (Bauerle et al., 2011). Another promising treatment is PBM using 670 nm red light to manage ischemic reperfusion injury. The therapeutic effects of PBM are thought to occur via the promotion of mitochondrial respiration by stimulation of cytochrome c oxidase to promote adenosine triphosphate (ATP) production and reduce oxidative stress (Karu, 1999; Silveira et al., 2009), although the precise mechanisms which enhanced mitochondrial function occurs remain unclear (Sommer, 2019). In neonatal rat models of hypoxia‐ischemia it has been found that PBM attenuated cognitive impairment, reduced brain injury and neuron loss, as well as prevented oxidative damage, mitochondrial fragmentation and cytochrome c release (Tucker et al., 2018; Yang et al., 2019; Yang et al., 2021). These studies were focused on neurological outcomes however and did not determine whether the kidney effects could also be improved by this treatment.

In this current study, strikingly, both treatments when given individually following HIE not only improved electron microscopic evidence of AKI and weight gain, but also ameliorated early onset elevations in NGAL and albumin, as well as persistent elevations in KIM‐1. This suggests they are effective at either rapid reversal of pathologic processes or prevention of injury development. Temporal changes in the urinary content of the biomarkers were varied, reflective of a progression in the activation of different pathological processes during AKI development. The transition away from processes that release early responding markers over time decreased differentiation between subjects with and without therapeutic treatments at longer time points. Albumin, for example, becomes elevated in the urine due to not only impaired renal reabsorption but expression of this gene is also injury‐inducible (Bolisetty & Agarwal, 2011; Ware et al., 2011). As kidney injury was detectable histologically 7 days following injury induction, a time when KIM‐1 remained elevated but NGAL and albumin elevations have resolved irrespective of treatment, this variation in urinary biomarker responses provides further support for the use of a panel consisting of multiple validated analytes. Because PBM is a promising therapy for AKI in neonates, any interaction with caffeine, which is frequently administered to treat apnea of prematurity (Alhersh et al., 2020), should be considered since they may be used concurrently. Furthermore, the half‐life of caffeine is prolonged in neonates (Aranda et al., 1979), so caffeine‐related interactions with PBM may occur even after caffeine administration is discontinued. Interestingly, when both PBM and caffeine where given in concert, their effectiveness at reversing HIE‐induced pathology and urinary biomarker change was somewhat diminished. As both caffeine and PBM can affect mitochondrial respiration (Dragicevic et al., 2012; Karu, 1999; Mishra & Kumar, 2014; Silveira et al., 2009; Vaughan et al., 2012), these activities may be counteractive and result in a loss in the therapeutic effects of this induction.

The kidney is a metabolically active organ, rich in mitochondria and is hemodynamically dependent on ATP to regulate water and solute balance. Processes that impair mitochondrial function, ATP production, and energy metabolism therefore can lead to cellular injury, negatively impacting kidney function. Mitochondrial dysfunction resulting in an inefficient use of oxygen for respiration has been associated with AKI in multiple disease settings, including in ischemia reperfusion injury (Clark & Parikh, 2020; Hall et al., 2013; Szeto et al., 2017). Analysis of mitochondrial respiratory chain complex activity revealed that HIE reduced of the flux of electrons between the mitochondrial respiratory chain complexes II and III, and that PBM and caffeine treatment also mirrored histological and urinary biomarker findings by reversing this electron chain defect more effectively when given individually than when administered as a combination treatment. It remains unclear why HIE did not have similar effects on the flux of electrons from complex I to III. It has been documented that PBM can directly stimulate mitochondrial respiratory chain complexes III and IV (Amaroli et al., 2016), which interact with each other to form respirasomes (Signes & Fernandez‐Vizarra, 2018). PBM may also increase their enzymatic activity through a mechanism where nitric oxide, which upon noncovalent binding to complex IV results in its inhibition, is photodissociated by absorption of a photon of red or near infrared light (Hamblin, 2018). Caffeine has also been found to interact with mitochondrial respiratory chain complexes, effecting the activity of complexes II, III, and IV to increase mitochondrial metabolism (Freddo et al., 2021; Verma et al., 2009). We can only speculate why the combination of PBM and caffeine ablated the beneficial effects of each intervention on complex II‐III electron activity. Perhaps the different mechanisms of action counteract each other at the level of the electron transport chain. However, a similar trend among the groups in tissue ultrastructure in Figure 2 suggests a link between the bioenergetic and structural pathology of the glomerulus, and it will be important to identify whether PBM and caffeine act on similar cell types within this structure and within other parts of the kidney.

Improving renal function in infants with HIE by protecting mitochondrial output has potential to positively impact both short‐term and long‐term recovery, however there remains much to be understood about the methodology through which these benefits occur. For example, to further understand how variation in the dynamics of treatment administration (i.e., treatment sequence, duration and interval between treatments) will affect outcomes. Additionally, directly exposing the kidneys to PBM requires sufficient penetration of the photons of light, which in humans may require more invasive approaches; however, there is a growing body of research that suggests that PBM when applied remotely to the organ of interest can exert beneficial effects through modulation of immune responses and secondary messenger signaling (Bian et al., 2022). In our study, the pups received a whole‐body exposure to PBM so we are not able to conclude whether the protective effects are due to direct or systemic mechanisms. The potential of off‐target application of PBM to provide benefit to less accessible tissues should be further explored as this could increase the feasibility of its use in the clinic in patients of all ages. Furthermore, our findings that combining treatments results in a reduction in benefit also highlight the limitations of use of these potential therapies without a more complete understanding of their mechanism of action.

## AUTHOR CONTRIBUTIONS

Groves et al. Effects of photobiomodulation and caffeine treatment on acute kidney injury in a hypoxic ischemic neonatal rat model. Angela M Groves: Conceived and designed research, performed experiments, analyzed data, interpreted results of experiments, prepared figures, drafted manuscript, edited and revised manuscript, approved final version of manuscript. Carl J Johnston: Conceived and designed research, performed experiments, interpreted results of experiments, edited and revised manuscript, approved final version of manuscript. Gisela Beutner: Performed experiments, analyzed data, interpreted results of experiments, prepared figures, edited and revised manuscript, approved final version of manuscript. Jane E Dahlstrom: Analyzed data, interpreted results of experiments, prepared figures, edited and revised manuscript, approved final version of manuscript. Mark E Koina: Performed experiments, analyzed data, interpreted results of experiments, prepared figures, approved final version of manuscript. Michael A O'Reilly: Conceived and designed research, approved final version of manuscript. Brian Marples: Interpreted results of experiments, edited and revised manuscript, approved final version of manuscript. George A Porter: Conceived and designed research, interpreted results of experiments, edited and revised manuscript, approved final version of manuscript. Patrick D Brophy: Conceived and designed research, interpreted results of experiments, approved final version of manuscript. Alison L Kent: Conceived and designed research, interpreted results of experiments, drafted manuscript, edited and revised manuscript, approved final version of manuscript.

## FUNDING INFORMATION

Partial financial support was received from NIH R01 HL144776, NIH R01 HL091968, and P30ES001247.

## CONFLICT OF INTEREST STATEMENT

None of the authors has any conflicts of interests.

## ETHICS STATEMENT

This article is original work that does not involve human subjects. All animal experimental protocols were approved by the University Committee on Animal Resources at the University of Rochester and were in accordance with the National Institutes of Health Guidelines.

## Supporting information


Figure S1:

Table S1:

File S1:

File S2:
