# Supplementary figures and images for: Effects of photobiomodulation and caffeine treatment on acute kidney injury in a hypoxic ischemic neonatal rat model
Source: Physiol Rep. 2023 Aug 7;11(15):e15773. doi: 10.14814/phy2.15773 (PMC10406568; doi:10.14814/phy2.15773)

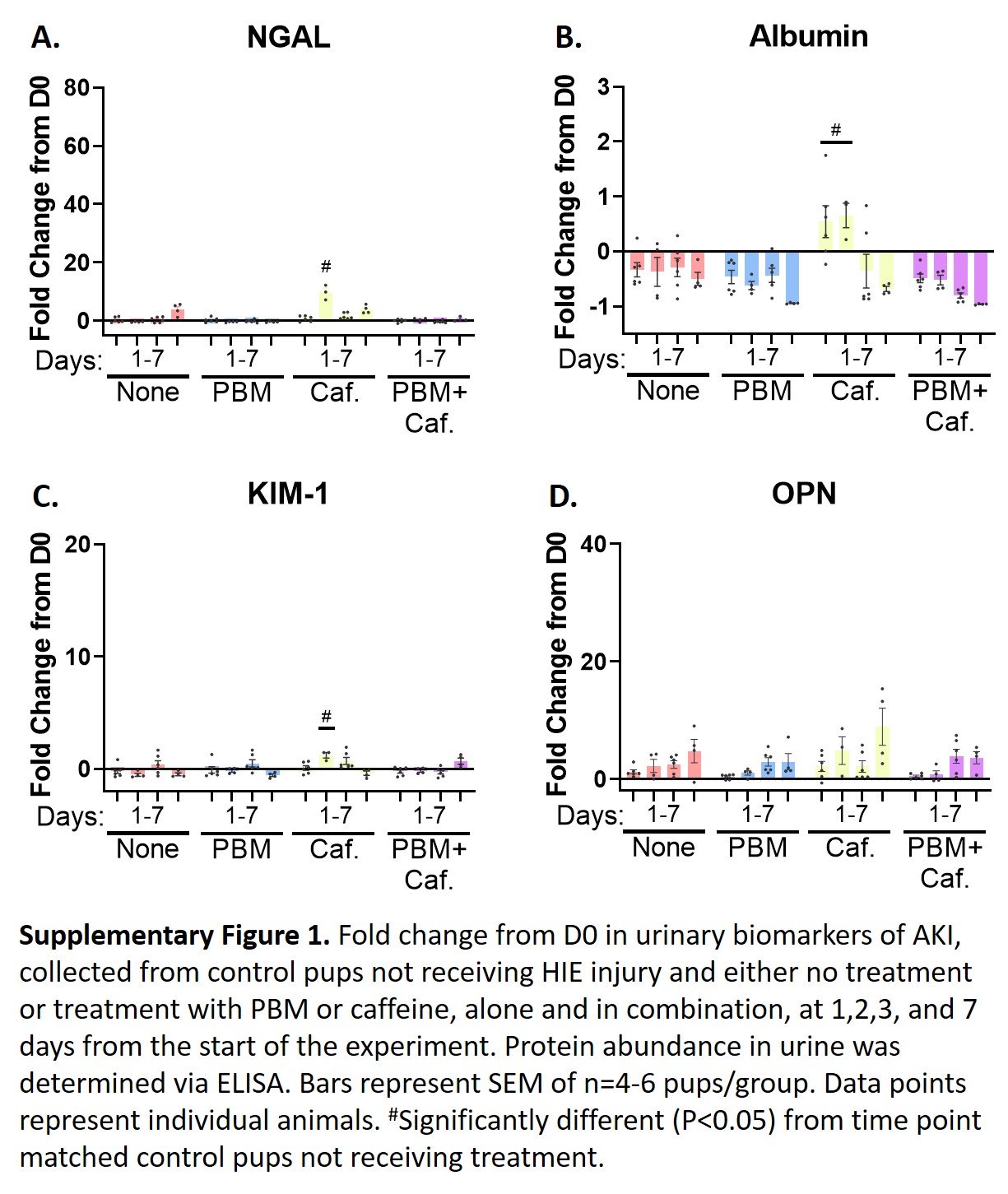

Supplement: Supplementary file 1 — Figure S1: [file PHY2-11-e15773-s004.jpg]
