# Supplementary material for: Effects of photobiomodulation and caffeine treatment on acute kidney injury in a hypoxic ischemic neonatal rat model
Source: Physiol Rep. 2023 Aug 7;11(15):e15773. doi: 10.14814/phy2.15773 (PMC10406568; doi:10.14814/phy2.15773)
Supplement: Supplementary file 2 — Table S1: [file PHY2-11-e15773-s002.docx]

Supplementary Table 1: Histopathology scores of Hematoxylin and Eosin stained right kidneys, evaluated on experimental day 7.

| Group: | Specimen: | Kidney: | Edema: | Grade of edema: | Tubules: | Proximal tubules dilated with ragged brush border: | Distal tubules dilated with ragged brush border: |
| --- | --- | --- | --- | --- | --- | --- | --- |
|  |  | **0=normal**  **X=abnormal** | **0=none**  **A=cortex**  **B=corticomedullary junction**  **C=medulla**  **D=B+C**  **E=all compartment** | **0=none**  **1=mild**  **2=moderate**  **3=severe** | **0=normal**  **X=abnormal** | **0=none**  **1=mild**  **2=moderate**  **3=severe** | **0=none**  **1=mild**  **2=moderate**  **3=severe** |
| None | N-1 | 0 | 0 | 0 | 0 | 0 | 0 |
|  | N-2 | 0 | 0 | 0 | 0 | 0 | 0 |
|  | N-3 | 0 | 0 | 0 | 0 | 0 | 0 |
|  | N-4 | 0 | 0 | 0 | 0 | 0 | 0 |
| HIE | H-1 | X | A | 1 | X | 3 | 2 |
|  | H-2 | X | B | 2 | X | 3 | 2 |
|  | H-3 | X | D | 2 | X | 3 | 2 |
| HIE  +  PBM | P-1 | X | B | 1 | X | 1 | 0 |
|  | P-2 | X | B | 1 | X | 1 | 0 |
|  | P-3 | X | B | 1 | X | 1 | 0 |
| HIE  +  Caffeine | C-1 | X | C | 1 | X | 3 | 1 |
|  | C-2 | X | D | 1 | X | 3 | 2 |
|  | C-3 | X | C | 1 | X | 3 | 1 |
| HIE  +  PBM + Caffeine | PC-1 | X | D | 1 | X | 2 | 0 |
|  | PC-2 | X | C | 1 | X | 2 | 0 |
|  | PC-3 | X | C | 1 | X | 3 | 0 |
