# Supplementary material for: Effects of photobiomodulation and caffeine treatment on acute kidney injury in a hypoxic ischemic neonatal rat model
Source: Physiol Rep. 2023 Aug 7;11(15):e15773. doi: 10.14814/phy2.15773 (PMC10406568; doi:10.14814/phy2.15773)
Supplement: Supplementary file 4 — File S2: [file PHY2-11-e15773-s003.pdf]

Effects of photobiomodulation (670nm red light) and caffeine treatment on acute kidney injury in a hypoxic ischemic neonatal animal model.

METHODS

Modified Rice Vannucci model of HIE

SD rat pups

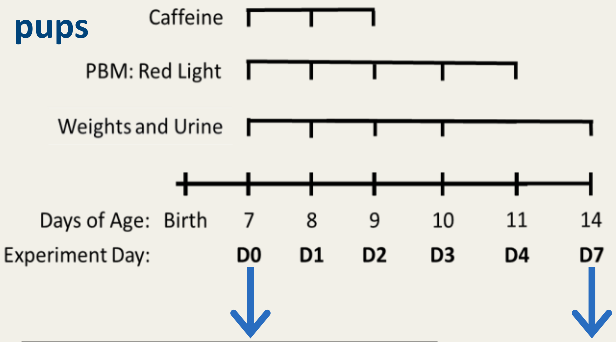

HIE:
- Ligation of left carotid artery
- 1 hr recovery with Dam
- 2 hr hypoxia (8% O_2)
- Return to Dam

Sample collection:
- Kidney
- Urine

OUTCOME

HIE:

Histological detection of kidney injury

Elevated urinary biomarker levels (NGAL, Albumin, KIM-1)

Decreased weight gain

Treatment with photobiomodulation or caffeine ameliorates AKI

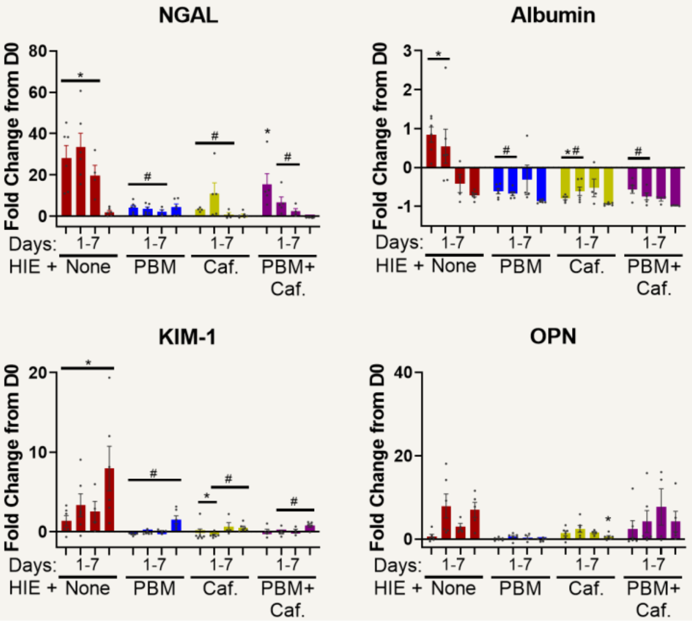

CONCLUSION Photobiomodulation and caffeine reduces AKI diagnosed by urinary biomarkers and confirmed by electron microscopy findings.
