# Supplementary material for: Effects of photobiomodulation and caffeine treatment on acute kidney injury in a hypoxic ischemic neonatal rat model
Source: Physiol Rep. 2023 Aug 7;11(15):e15773. doi: 10.14814/phy2.15773 (PMC10406568; doi:10.14814/phy2.15773)
Supplement: Supplementary file 3 — File S1: [file PHY2-11-e15773-s001.docx]

Josephine C. Adams, M.A, Ph.D.	7/5/2023

Editor-in-Chief, Physiological Reports

University of Bristol

Bristol, UK

Dear Josephine,

I eagerly submit to Physiological Reports the revisions to our article entitled:

**Effects of photobiomodulation and caffeine treatment on acute kidney injury in a hypoxic ischemic neonatal rat model.**

The motivation for the study arose from recently published observations that a frequently utilized animal model of hypoxic ischemic encephalopathy, the Rice-Vannucci neonatal rat model, also recapitulates the acute kidney injury that is a common component perinatal asphyxia. In this manuscript we longitudinally tracked the progression of AKI using urinary biomarkers of acute kidney injury and demonstrated that treatment of pups with either photobiomodulation or caffeine reduced kidney injury. Furthermore, our results indicate that these outcomes may be mediated by effects on mitochondrial respiration. We thank the editor for their comments, which we made our best efforts to address. We hope that this has improved the manuscript such that it is now acceptable for publication.

The data in this manuscript are original and have not been published previously. The manuscript is not currently under consideration by any other journal. All authors have read and approved the final version of this manuscript.

Funding: Partial financial support was received from NIH R01 HL144776, NIH R01 HL091968, and P30ES001247.

Competing interests: The authors have no relevant financial or non-financial interests to disclose.

Please do not hesitate to contact me if you have any further questions.

Angela M Groves Ph.D.

Research Assistant Professor

Department of Radiation Oncology

University of Rochester Medical Center

601 Elmwood Avenue

Rochester, New York, 14642
